# Anterior Tibial Tendon Transfer for Relapsed and Residual Clubfoot Deformity and Functional Outcome With Timely Intervention: How Have We Done Over the Last Five Years?

**DOI:** 10.7759/cureus.110856

**Published:** 2026-06-14

**Authors:** Irfan Ahmad, Rana Dilawaiz Nadeem, Muhammad Ayaz Ul Haq Chatta

**Affiliations:** 1 Trauma &amp; Orthopaedics Surgery, King's College Hospital, London, GBR; 2 Trauma &amp; Orthopaedics Surgery, Rashid Latif Khan University (RLKU) Medical College, Lahore, PAK; 3 Trauma &amp; Orthopaedics Surgery, Navan Regional Orthopaedic Unit, Our Lady's Hospital, Navan, IRL

**Keywords:** achilles tenotomy, clubfoot, ponseti method, recurrence, tibialis anterior tendon transfer, treatment outcome

## Abstract

Objective: To evaluate the functional and clinical outcomes of anterior tibial tendon transfer in patients with relapsed or residual clubfoot deformity presenting with dynamic supination and forefoot adduction after treatment with the Ponseti method.

Materials and methods: A retrospective multicentre review was conducted on patients with idiopathic clubfoot treated by the Ponseti method who subsequently developed relapse with forefoot adduction and dynamic supination between January 2019 and January 2024 at Mayo Hospital and Rashid Latif Khan University, Lahore, Pakistan. All procedures were performed by a single surgeon. Forty patients (55 feet) underwent anterior tibial tendon transfer; 23 feet (41.8%) additionally required Achilles tenotomy. Mean age at surgery was 6.2 ± 2.5 years, and mean follow-up was 3.2 ± 1.5 years. Functional and clinical outcomes, along with parental satisfaction, were assessed.

Results: Among 40 patients (55 feet), 26 (65%) were males and 14 (35%) females; 15 (55%) had bilateral and 25 (45%) unilateral deformities. Mean preoperative score improved from 89.8 ± 12.3 to 134.4 ± 10.5 postoperatively (p < 0.005). Postoperative outcomes were excellent in 45 (81.8%) feet and good in 10 (18.2%) feet. All patients demonstrated improvement in functional grading.

Conclusion: Anterior tibial tendon transfer resulted in significant improvement in functional outcomes and correction of residual dynamic clubfoot deformity. Early intervention may help achieve correction before the development of rigid deformity.

## Introduction

Club foot has an incidence of up to seven per 1,000 live births [1]. The Ponseti method of serial casting, manipulation, and bracing has resulted in painless, plantigrade, and well-aligned feet in long-term studies [2]. However, residual deformities resulting in dynamic supination and adduction of the forefoot have been reported up to 56% after the Ponseti method [3,4]. Rarely, these may correct spontaneously with maturation without any intervention; however, if not resolved, they may result in residual dynamic supination and adduction of the forefoot, leading to functional gait disturbance and shoe-fitting problems [4].

Literature for the cause of this relapse supports different theories; it can be due to non-compliance and inadequate use of the abduction brace [4-6]. Another theory supports the muscle imbalance contributing towards this deformity, with overpowering tibialis anterior (TA) and weak peronei. Farsetti et al. noted forefoot supination deformity after peronei transfer in paralytic foot [7]. Transfer of the TA to the lateral and dorsum of the foot has been used by many authors to correct this deformity with significant results [8-10]. 

The current study was conducted to analyse the functional outcome and correction of deformity by anterior tibial tendon transfer in dynamic supination and forefoot adduction deformity after clubfoot treatment by Ponseti.

## Materials and methods

Study design and setting

This retrospective multicentre study included patients with idiopathic clubfoot previously treated with the Ponseti method who subsequently developed residual or relapsed deformity characterized by dynamic supination and forefoot adduction. The study was conducted at the Department of Orthopaedic Surgery, Mayo Hospital, Lahore, and the Department of Orthopaedics, Rashid Latif Khan University, Lahore, Pakistan. Patients managed between January 2019 and January 2024 were identified, and data collection was performed from June to August 2024 after obtaining institutional ethical approval.

Patient selection

Patients with residual or relapsed clubfoot deformity following treatment with the Ponseti method who underwent anterior tibial tendon transfer were included in the study. The decision for surgical intervention was based on clinical assessment demonstrating dynamic supination with forefoot adduction, associated functional limitations, difficulty with brace compliance, and parental dissatisfaction regarding deformity correction.

Patients with neuromuscular disorders, congenital syndromic conditions, previous soft tissue or bony surgical procedures, and patients with additional recurrent deformities requiring alternative procedures were excluded.

A total of 40 patients (55 feet) met the inclusion criteria and underwent anterior tibial tendon transfer. There were 26 male and 14 female patients. Bilateral deformity was present in 15 patients, while 25 had unilateral involvement. The mean age at surgery was 6.2 ± 2.5 years. In addition to tendon transfer, 23 feet (41.8%) required concomitant Achilles tendon tenotomy.

Surgical procedure

All procedures were performed by a single surgeon under general anaesthesia with the patient in the supine position on a standard operating table.

The tibialis anterior tendon insertion was identified at the medial cuneiform and base of the first metatarsal through a small medial incision. Following identification and confirmation of the tendon, it was detached from its insertion and secured using a stay suture. The tendon was mobilized proximally while maintaining its course beneath the extensor retinaculum to preserve normal biomechanics and reduce bowstringing.

A second incision was then made over the dorsolateral aspect of the foot. A subcutaneous tunnel was created to transfer the tendon laterally. The tendon was transferred and fixed through a drill hole created in the lateral cuneiform aligned with the base of the third metatarsal, allowing a more balanced dorsiflexion vector and correction of dynamic supination. The foot was maintained in neutral or slight dorsiflexion with eversion during fixation. The transferred tendon was secured using pull-out sutures tied over a sterile button and protective felt padding on the plantar surface. The suture used was a nonabsorbable nylon suture, and a knot over the button was made.

Patients exhibiting equinus on the operating table after anaesthesia with inability to passively dorsiflex the ankle beyond neutral position underwent Achilles tenotomy with a stab incision.

Following wound closure, patients were immobilized in a weight-bearing short-leg cast for six weeks in the neutral position of the foot. The patient was allowed weight-bearing mobilization in the cast. After six weeks, no formal physiotherapy was required.

**Figure 1:**
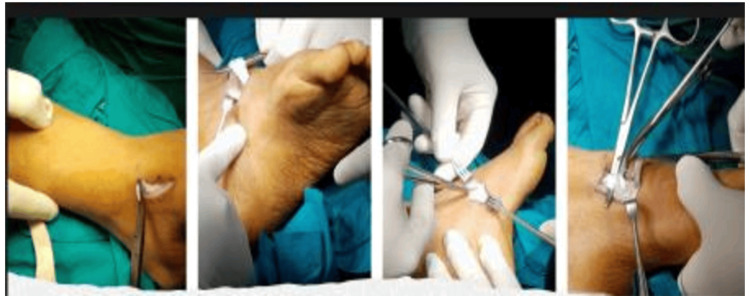
Surgical incision over tibialis anterior tendon.

**Figure 2:**
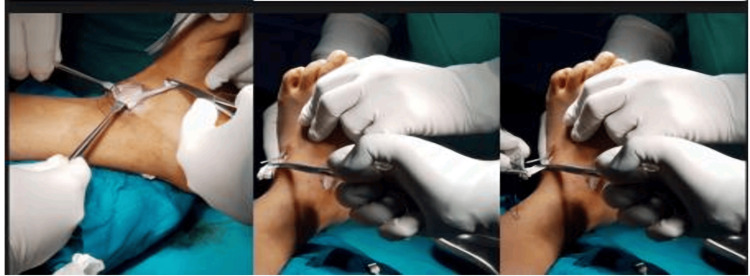
Tendon harvesting and dorsal foot incision.

**Figure 3:**
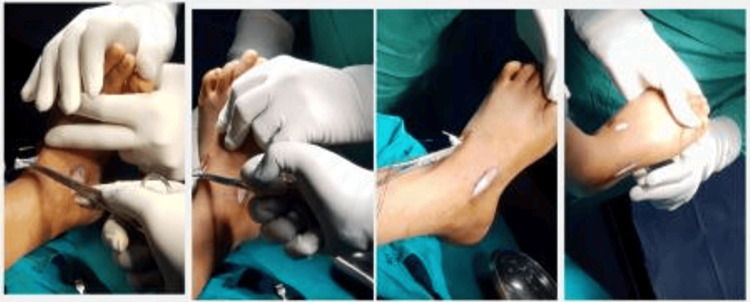
Tendon transfer and fixation technique.

**Figure 4:**
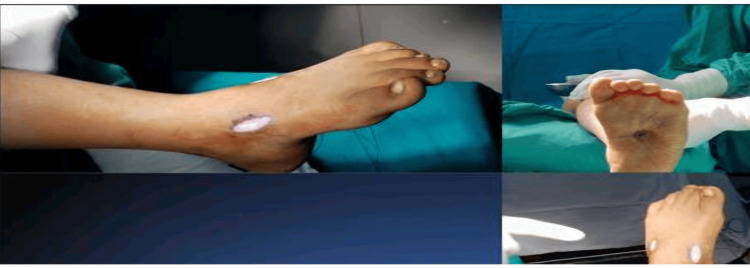
Final correction.

Representative intraoperative steps, including tendon identification, tendon harvesting, and tendon transfer, are demonstrated in Figures 1-4.

Follow-up and outcome assessment

The patients underwent regular postoperative follow-up with clinical and functional assessment. The postop score was calculated at six months of follow-up. Although the patients were followed up at two weeks, six weeks, one year, two years, and five years, not all patients showed up at five year final follow-up. The mean follow-up duration was 3.2 ± 1.5 years.

Outcomes were assessed using a modified functional rating system derived from previously published clubfoot evaluation tools. The assessment included functional and clinical parameters, as well as parental satisfaction regarding deformity correction, shoe wear, and mobility.

Functional outcomes were categorized as follows: Excellent: 130-150, Good: 110-129, Fair: 90-109, and Poor: < 90.

Statistical analysis

Statistical analysis was performed using Statistical Product and Service Solutions (SPSS, version 22.0; IBM SPSS Statistics for Windows, Armonk, NY). Continuous variables were expressed as mean ± standard deviation (SD), whereas categorical variables were presented as frequencies and percentages.

Normality of continuous variables was assessed using the Shapiro-Wilk test together with visual inspection of histograms. Functional score data demonstrated approximate normal distribution (Shapiro-Wilk W = 0.978, p = 0.27). Therefore, preoperative and postoperative functional scores were compared using a paired-samples t-test.

Statistical analysis of functional outcomes was performed on a per-foot basis (n = 55), as deformity correction and functional grading were assessed individually for each operated foot, whereas demographic characteristics were summarized on a per-patient basis (n = 40).

Cases with incomplete records or missing outcome data were excluded during data collection; therefore, no imputation method for missing data was required. Statistical significance was defined as a two-sided p-value < 0.001.

## Results

Patient demographics and clinical characteristics

A total of 40 patients (55 feet) underwent anterior tibial tendon transfer for relapse deformity following treatment with the Ponseti method. There were 26 (65%) males and 14 (35%) females, with a male-to-female ratio of 1.8:1. Bilateral involvement was observed in 15 (37.5%) patients, while 25 (62.5%) had unilateral deformities. Of the 55 operated feet, 35 (63.6%) were left-sided, and 20 (36.4%) were right-sided. The mean age at the time of surgery was 6.2 ± 2.5 years (range: 4-8.5 years). The mean follow-up duration was 3.2 ± 1.5 years. Additionally, 23 (41.8%) feet underwent concomitant Achilles tendon tenotomy.

Functional outcomes

Preoperatively, according to the functional rating system, 29 (52.7%) feet were graded as fair and 26 (47.3%) as poor. Postoperatively, outcomes improved substantially, with 45 (81.8%) feet achieving excellent grades and 10 (18.2%) demonstrating good outcomes. No feet remained in the fair or poor categories after surgery. All patients showed improvement by at least two functional grades. Detailed functional grade distributions before and after surgery are summarized in Table 1.

**Table 1:** Number of operated feet (n = 55) in each functional grade, with percentages in parentheses.

Functional grade score	Preoperative (n=number of feet)	Postoperative at 6 months (n=number of feet)
Excellent (130-150)	0	45 (81.8%)
Good (110-129)	0	10 (18.2%)
Fair (90-109)	29 (52.7%)	0
Poor (<90)	26 (47.3%)	0

Normality testing using the Shapiro-Wilk test demonstrated approximately normal distribution of functional score data (W = 0.978, p = 0.27); therefore, a paired-samples t-test was used for comparison of preoperative and postoperative functional scores. Statistical analysis was performed on a per-foot basis (n = 55), whereas demographic characteristics were summarized on a per-patient basis (n = 40).

The mean functional score improved significantly from 89.8 ± 12.3 (range: 68-113) preoperatively to 134.4 ± 10.5 (range: 111-150) postoperatively (paired t-test: t = 21.84, df = 54, p < 0.001). Detailed functional score outcomes are summarized in Table 2.

**Table 2:** Mean preoperative and postoperative functional sub-scores of operated feet (n = 55). Values represent the mean scores per parameter (maximum possible score in brackets), averaged across all feet. Total score = sum of parameters.

Parameter	Maximum score	Preoperative Mean ± SD	Postoperative Mean ± SD
Ankle dorsiflexion (passive)	>90° = 10, 90° = 05, <90° = 00 (max = 10)	5.8 ± 2.1	9.2 ± 1.1
Subtalar joint (passive)	>15° = 10, <15° = 5, Stiff = 0 (max = 10)	7.7 ± 1.8	8.1 ± 1.0
Heel position (standing)	0-5 valgus = 10, >5 valgus = 5, varus =0 (max = 10)	6.2 ± 1.5	9.6 ± 0.8
Forefoot appearance	Neutral = 10, <5 add/abd = 5, >5 add/abd = 0 (max = 10)	4.1 ± 1.4	9.4 ± 0.7
Supination	Absent = 10, present = 0 (max = 10)	2.5 ± 1.8	9.7 ± 0.5
Cavus	Absent = 10, present = 0 (max = 10)	4.0 ± 1.7	9.3 ± 0.6
Gait	Normal = 20, heel gait = 10, toe gait = 05 (max = 20)	16.0 ± 2.4	16.0 ± 1.5
Shoe type	Regular = 5, special = 0 (max = 5)	2.7 ± 0.8	5.0 ± 0.0
Functional limitation	None = 20, occasional = 15, frequent = 05 (max = 20)	17.5 ± 2.1	18.5 ± 1.4
Pain	Never = 20, occasional = 15, frequent = 05 (max = 20)	15.0 ± 2.0	18.0 ± 1.1
Patient satisfaction	High = 20, partial = 10, not satisfied = 0 (max = 20)	4.3 ± 1.2	16.8 ± 0.7
TA tendon function	Pronation and dorsiflexion = 5, mainly dorsiflexion = 3, mainly supination = 0	4.0 ± 0.9	4.8 ± 0.3
Total score	150	89.8 ± 12.3	134.4 ± 10.5

Clinical and functional outcomes

Clinical and functional assessment during follow-up demonstrated correction of dynamic supination and forefoot adduction in all operated feet. Improvements in gait pattern, shoe wear, mobility, and overall function were observed postoperatively, and parents reported high satisfaction regarding cosmetic appearance and functional improvement. 

Complications and follow-up outcomes

The mean duration of follow-up was 3.2 ± 1.5 years. During follow-up, no recurrence or overcorrection was observed. Two patients (5%) developed superficial wound infections, which resolved with oral antibiotics and local wound care without further complications. No patient required revision surgery or experienced major postoperative complications.

Till the last follow-up of our patients, no patient had a recurrence of deformity. Though our scores postoperatively were recorded at six months of follow-up, there was no worsening of clinical and functional outcome at subsequent follow-ups.

## Discussion

Forefoot dynamic supination deformity was documented after the Ponsetti method and consists of both forefoot adduction and dynamic supination. An important consideration when planning treatment is differentiating between dynamic and rigid deformities, as rigid deformities may result from metatarsus varus, an undercorrected talonavicular joint, soft tissue contractures, or bony abnormalities [11].

The progression of dynamic supination has been investigated and postulated that weak peronei and overpowering TA tendon are the main cause, the imbalance, if left untreated, can even result in fixed deformity [12,13], and a tendon balancing procedure is the right road towards the treatment [14]. In 1940, Garceau et al. reported anterior tibial tendon transposition [8,9]; since then, it has become an important part of management [15]. This previously described method by Garceau et al. [8] to the lateral side of the foot (cuboid, fifth metatarsal base) resulted in overcorrection [16,17].

In our study, transfer of the tendon in line with the third metatarsal base and to the lateral cuneiform did not result in overcorrection in even a single patient; that is why, in our study, we concluded that transferring to the lateral cuneiform improves dorsiflexion and deformity of the forefoot without any overcorrection or undercorrection. The timing of the surgery has not been defined in the literature, but the majority of studies conducted have an age limit between four and eight years. In our study, the average age at the time of surgery was 6.2 ± (2.5) years. We have achieved excellent-to-good postoperative results in all our cases using the modified functional score [17-19]. In our study, 45 patients had excellent postoperative scores, and 10 patients had good scores, with 89.8 ± 12.3 (range: 68-113) preoperatively to 134.4 ± 10.5 (range: 111-150) postoperatively and with a significant p-value (< 0.001). In our study, all the parameters improved postoperatively; no patient required revision surgery or recurrence, which shows the excellent results of TA tendon transfer at an early age. Our results are consistent with the study conducted by Ezra et al., in which all the patients scored excellent and good postoperatively using the split and full tibialis tendon transfer in both groups. Additionally, our results are consistent with the studies by Ezra et al., Holt et al., and Kuo et al. [3,20-23].

Functional parameters evaluated by surgeons, parents, and patients justify this procedure. In our study, we paid particular importance to the patient's subjective improvement and parents' satisfaction with the surgery results.

Our study showed that, apart from significant improvement in dynamic supination and forefoot adduction, there was also an improvement in ankle and subtalar joint movement postoperatively, as shown by Kuo et al. [3].

Garceau et al. [8,9] originally passed the tendon under the annular ligament; however, we prefer a subcutaneous route to avoid adhesions, as it provides a better range of motion and power. A potential drawback of the subcutaneous transfer is the development of a bowstring effect at the ankle joint.

Our technique required only two incisions, making it less invasive; however, the literature [22] describes techniques involving more than two incisions. We did not encounter any difficulties in tendon harvesting using the two-incision approach, which we found to be effective and less invasive.

Overall, our data show that anterior tibial tendon transfer is an excellent modality to correct residual dynamic clubfoot deformity. The procedure should ideally be performed between four and eight years of age, as intervening earlier than four years or later than eight years may be less optimal due to progressive stiffening of the deformity over time.

This study has a few limitations. First, its retrospective design introduces the potential for selection and information bias. Second, the study included a relatively small sample size from two institutions, which may limit the generalizability of the findings. Third, all procedures were performed by a single surgeon; while this ensured consistency in surgical technique and postoperative management, it may reduce the external applicability of the results. In addition, no comparative group was included, preventing direct comparison with alternative treatment strategies. Finally, although functional outcomes and parental satisfaction were assessed, longer follow-up would be useful to evaluate the durability of correction and identify any late recurrence or overcorrection. Future prospective studies with larger cohorts and comparative analysis are required to further validate these findings.

## Conclusions

Anterior tibial tendon transfer is an effective procedure for the management of residual and relapsed dynamic clubfoot deformity following treatment with the Ponseti method. In our series, the procedure resulted in significant improvement in functional scores, correction of dynamic supination and forefoot adduction, improved gait pattern, better shoe wear, and high parental satisfaction, with a low complication rate and no recurrence during follow-up. The findings support the concept that muscle imbalance, particularly overpowering tibialis anterior function in the presence of weak peronei, plays an important role in relapse deformity. Timely surgical intervention before the development of fixed or rigid deformity may help avoid the need for more extensive soft tissue or bony procedures. Anterior tibial tendon transfer, when performed with appropriate patient selection and surgical technique, remains a reliable and reproducible option for achieving a plantigrade, functional, and cosmetically improved foot in children with recurrent clubfoot deformity.
